# Clinical outcomes of dupilumab therapy in chronic rhinosinusitis with nasal polyps in a Canadian tertiary care rhinology practice

**DOI:** 10.1186/s13223-023-00782-7

**Published:** 2023-03-30

**Authors:** Elysia Grose, Alyssa Y. Li, John M. Lee

**Affiliations:** Division of Rhinology, Department of Otolaryngology-Head and Neck Surgery, University of Toronto, 600 University Avenue, Toronto, ON M5G 1X5 USA

**Keywords:** Biologics, Chronic rhinosinusitis, Endoscopic sinus surgery, Sinonasal outcomes, Asthma

## Abstract

**Background:**

In 2020, dupilumab became the first monoclonal antibody therapy to be approved by Health Canada for the treatment of chronic rhinosinusitis with nasal polyps (CRSwNP). The primary aim of this study was to characterize the outcomes in an initial cohort of patients with CRSwNP who have undergone dupilumab therapy.

**Methods:**

A retrospective study was conducted of patients with CRSwNP who were treated with dupilumab. Demographic information, comorbidities, number of previous surgeries, and insurance information were collected. The primary outcome were changes in the sinonasal outcome test (SNOT-22) scores from baseline to timepoints after receiving dupilumab.

**Results:**

Forty-eight patients were considered for dupilumab therapy, and 27 (56%) received coverage or were able to fund the medication independently. Patients waited an average of 3.6 months to obtain access to the medication. The mean age of the patients was 43. Forty-one percent (11/27) of patients had aspirin exacerbated respiratory disease, and 96% (26/27) had a diagnosis of asthma. The mean length of time on dupilumab was 12.1 months. The baseline SNOT-22 score was 60.6. The mean decrease at 1 month, 3 months, 6 months, and 12 months after starting dupilumab was 8.8, 26.5, 42.8, and 33.8, respectively. There were no serious adverse events.

**Conclusion:**

Patients treated with dupilumab in a Canadian tertiary care rhinology clinic demonstrated substantial clinical improvement as measured by disease-specific sinonasal outcomes. Further studies are needed to determine the longer-term effectiveness and adverse event profile of this novel therapy.

## Introduction

Chronic rhinosinusitis with nasal polyps (CRSwNP) is characterized by inflammation of the nasal mucosa and paranasal sinuses, and affects 6 to 12% of people in the Western world [1, 2]. Surgical management, namely functional endoscopic sinus surgery (FESS), has enhanced the care of patients with medically refractory CRSwNP, however, surgical reintervention or revision is often necessary in up to 30% of patients [3]. The risk of disease recurrence and revision surgery is even higher among the 26% and 48.3–55% of patients with CRSwNP and co-morbid aspirin exacerbated respiratory disease (AERD) and asthma, respectively. [4–7] While the precise pathophysiology of CRSwNP has yet to be fully elucidated, eosinophilic inflammation and polyclonal activation of type 2 helper T cells have been demonstrated in CRSwNP, leading to the release of inflammatory cytokines, including interleukin 4 (IL-4), IL-5, and IL-13 [8–10]. As such, biologic agents, which target and reduce type 2 inflammation, have since been developed and are surfacing as a novel treatment for patients with refractory CRSwNP.

Dupilumab is an anti-IL-4 and IL-13 monoclonal antibody that has emerged as a novel treatment modality for patients with medically refractory CRSwNP. In the SINUS-24 (NCT02912468) and SINUS-52 (NCT02898454) phase 3 randomized controlled trials, dupilumab was found to significantly improve clinical and radiologic signs of CRSwNP in patients [11–13]. Improvements in smell, clinical status based on the sino-nasal outcome test (SNOT-22), and health-related quality of life were similarly identified across patients with CRSwNP [11, 12]. Following its approval by Health Canada in August 2020, dupilumab has been recommended for the treatment of Canadian patients with CRSwNP and prior functional endoscopic sinus surgery and medical therapy [14]. Since then, short-term results have been reported in Canadian patients who meet this criteria; among these patients, the most significant benefits to quality of life were seen in rhinologic symptoms, sleep quality, and mental health [15]. In Canada, dupilumab is not routinely covered by universal health insurance and may be only covered by private insurers in some circumstances. With an annual out-of-pocket cost exceeding $25,000–30,000, there is a clear need to rigorously evaluate the indications for this novel treatment and its outcomes to guide patients toward the most efficacious and economic therapies [14]. The primary aim of this study was to characterize the outcomes in an initial cohort of patients with CRSwNP who have undergone dupilumab therapy in a Canadian tertiary care rhinology practice.

## Methods

### Study design and patient population

Approval of this study was granted by the Unity Health Toronto Research Ethics Board at St. Michael’s Hospital, Toronto, Ontario, Canada (REB No. 20-231). A retrospective review of all patients with a diagnosis of CRSwNP, who underwent treatment with dupilumab in the Rhinology Clinic at St. Michael’s Hospital in Toronto, Ontario from September 1, 2020 to May 1, 2022, was conducted. All patients included in the study received a diagnosis of CRSwNP as per guidelines of the Canadian and American Academies of Otolaryngology-Head & Neck Surgery [16, 17]. Patients were excluded if they had underlying cystic fibrosis, primary ciliary dyskinesia, primary immunodeficiencies (such as common variable immunodeficiencies), uncontrolled human immunodeficiency virus infection, or if they were < 18 years of age at the time of initial consultation.

The treatment of CRSwNP at our institution includes medical and surgical therapy. At the time of initial consultation, all patients underwent computed tomography (CT) imaging of the sinuses, which was graded using the Lund-Mackay CT scoring system. All patients received maximal medical therapy, including nasal saline irrigation, intranasal and systemic corticosteroids, and antibiotics. Patients with medically refractory disease were offered surgical therapy initially, including FESS (minimum Draf IIa; opening of all four paranasal sinuses bilaterally) with or without concurrent septoplasty, endoscopic modified Lothrop procedure (Draf III), and/or in-office polypectomy, which were performed by the study’s senior author (J.M.L.). Biologics were discussed with patients who had not had surgery if they were medically unable to undergo surgery or preferred medical management. Regular follow-up visits were scheduled at 1-, 3-, 6-, and 12-month intervals, or sooner as clinically required. During each visit, patients completed a SNOT-22 questionnaire. The SNOT-22 questionnaire is a validated test designed to measure disease-specific health related quality of life for CRS. It consists of 22 items rated from 0 (“no problem at all”) to 5 (“worst possible symptom”) with total scores ranging from 0 to 110 with higher SNOT-22 total corresponding to worse symptoms. The minimum clinically important difference for SNOT-22 scores is 8.9 [18].

### Outcome measurements and data collection

Electronic medical records were accessed to collect patient demographic data, including gender, age, baseline Lund-Mackay CT score, previous treatment history, medical therapies, date of previous surgery, and type of surgery. Dupilumab start date, dose, frequency, and insurance coverage status were recorded. Dupilumab start date and date of last documented clinical encounter were used to calculate follow-up time. Baseline and 1-, 3-, 6-, and 12-month post-dupilumab SNOT-22 scores were also collected retrospectively.

### Data analysis

Descriptive statistics were used to summarize the characteristics of the overall cohort. Categorical variables were summarized by expressing the frequency and proportion while continuous variables were summarized by reporting the mean and standard deviation (SD). All analyses were performed using the statistical software, R (version 3.4.3, R Foundation for Statistical Computing, Vienna, Austria).

## Results

Forty-eight patients were considered for dupilumab therapy, and 27 (56%) received coverage or were able to fund the medication independently. Three patients (3/27, 11%) paid out of pocket for the medication, whereas the remaining 24 (24/27, 89%) received coverage from a private insurance company. Thus, 27 patients were currently undergoing dupilumab therapy for CRSwNP and were included in the analysis. The 27 patients approved for dupilumab waited on average 3.6 months (SD: 3.5) to obtain access to the medication. The baseline characteristics of the included cohort are detailed in Table 1. Ninety three percent of the included patients had one or more previous sinus surgery. For the two patients who did not have sinus surgery, they were both offered surgery and it was patient preference to trial dupilumab instead of any surgical intervention. All patients had previously tried intranasal corticosteroids and at least one course of oral corticosteroids.

**Table 1:** Patient demographics

Characteristic	
Age (years), mean ± SD	43 ± 10.9
Male, n (%)	15 (55.6)
AERD, n (%)	11 (40.7)
Asthma, n (%)	26 (96.3)
Number of previous sinus surgeries	
0	2
1	8
2	7
≥ 3	10
Baseline Lund-MacKay Score, mean ± SD	18.7 ± 4.9
Length of time on dupilumab (months), mean ± SD	12.1 ± 5.0
Time from last surgery to starting dupilumab (months), mean ± SD	31.1 ± 25.4
Total follow up time (months), mean ± SD	10.8 ± 5.1

*AERD* aspirin exacerbated respiratory disease, *SD* standard deviation

**Fig. 1:**
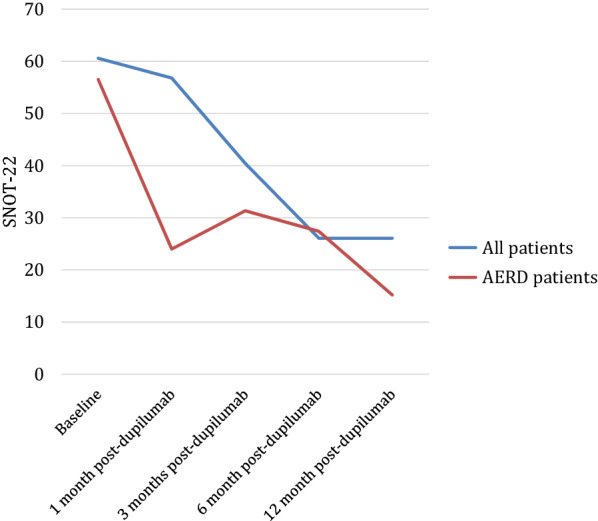
SNOT-22 scores

During the study period, two patients discontinued therapy due to adverse effects. One patient stopped the medication due to pain at the injection site and another patient stopped the medication due to the development of lower leg edema. No other adverse reactions were encountered. The same patient who terminated dupilumab due to new leg edema required revision FESS after stopping the medication. The remainder of the included patients did not require any sinus surgeries after starting dupilumab. The SNOT-22 results before and after starting dupilumab are depicted in Fig. 1 and Table 2.

**Table 2:** SNOT-22 outcomes

	Baseline SNOT-22	SNOT-22 1-month post-dupilumab	Change in SNOT-22 1-month post-dupilumab	SNOT-22 3 months post-dupilumab	Change in SNOT-22 3-months post-dupilumab	SNOT-22 6 months post-dupilumab	Change in SNOT-22 6-months post-dupilumab	SNOT-22 12 months post-dupilumab	Change in SNOT-22 12-months post-dupilumab
Total cohort (Mean ± SD)	60.6 ± 18.8	56.8 ± 37.4	− 8.8 ± 10.5	40.4 ± 16.8	− 26.5 ± 14.0	26.1 ± 22.0	− 42.8 ± 24.6	26.1 ± 17.9	− 33.8 ± 26.3
AERD (Mean ± SD)	56.5 ± 14.4	24 ± 33.9	− 5.5 ± 7.8	31.3 ± 22.2	− 28.3 ± 16.2	27.4 ± 21.8	− 40.3 ± 29.0	15.2 ± 17.8	− 46.0 ± 18.9

All values are reported as means ± standard deviations

*SNOT-22* Sinonasal outcome test, *SD* standard deviation, *AERD* aspirin exacerbated respiratory disease

## Discussion

Dupilumab has been shown to significantly reduce polyp size, sinus opacification, enhance patient quality of life, and is now being incorporated as a part of the standard of care for patients with CRSwNP [11]. Moreover, the Canadian Rhinology Working Group recommends that patients be considered for biologic therapy if they have either failed FESS and standard medical therapy or if they are incapable of undergoing FESS or medical therapy [14]. Thus, there is a need to better understand the clinical outcomes associated with dupilumab therapy in a real world, Canadian healthcare context. In this series, we present the clinical characteristics and quality of life outcomes of patients on dupilumab in a tertiary care rhinology practice in Toronto, Ontario, Canada.

In the Canadian setting, one of the main barriers to accessing dupilumab therapy is obtaining private insurance coverage since the medication is not routinely covered by provincial drug programs [14, 15]. The estimated annual cost of dupilumab approaches $30,000 CAD [19]. This is in contrast to the estimated $3,500 CAD that it costs the Canadian healthcare system to perform FESS [20]. Although the Canadian Agency for Drugs and Technologies (CADTH) recommends reimbursing patients for dupilumab when used for severe asthma, they have yet to publish reimbursement recommendations for the medication for CRSwNP [21]. Thus, patients must endure lengthy approval processes with their private insurance companies or pay out of pocket, making access to this medication financially and logistically challenging despite financial assistance programs. In this study, just over half of the patients considered for dupilumab for CRSwNP received approval from their insurance providers and were subsequently initiated on therapy. The patients who did begin therapy waited an average of 3.6 months to obtain access and receive funding for the medication from the time they were prescribed the medication. These findings are not dissimilar to another recent study conducted in Canada, in which 49% of patients considered for dupilumab therapy received coverage and started therapy, further demonstrating the accessibility challenges that exist within the Canadian context for dupilumab [15].

The SNOT-22 score is a widely used disease-specific quality of life outcome that is able to reliably discern treatment response [22]. Treatment with dupilumab has been demonstrated to improve SNOT-22 scores beyond a mean difference of 8.9, or the minimal difference of clinical importance, in two randomized control trials and another Canadian retrospective study [11, 15]. The mean baseline score recorded in our cohort was 60.6, which is comparable to the baseline scores for patients undergoing dupilumab therapy in SINUS-52 (baseline SNOT-22 score of 51) and a recently conducted Canadian retrospective study (baseline SNOT-22 score of 61), indicating patients placed on dupilumab in these studies experienced comparable baseline quality of life symptoms. In this study, we found a 42.8 and 33.8 point reduction in SNOT-22 scores at 6 and 12 months respectively. This is similar to the changes found in the SINUS-52 randomized control trial which found a change of 28 points and 30 points respectively at approximately 6 and 12 months [11]. Additionally, the current study demonstrates the gradual improvement of sinonasal symptoms and quality of life with incrementally increasing mean changes seen at 1, 3, 6, and 12 months (mean change: 8.8, 26.5, 42.8, and 33.8, respectively). Similar SNOT-22 data from the early months after starting dupilumab is seldom seen in the literature despite its importance in counseling patients about their expected response to therapy [23].

Dupilumab has also been shown to reduce intranasal corticosteroid use and reduce the need for FESS [11, 24]. A randomized control trial conducted by Bachert et al. found that only 1.5% of patients with CRSwNP and a history of prior sinus surgery who were taking dupilumab and mometasone spray required FESS after 1 year compared to 11.4% who were taking mometasone spray alone [11]. Similarly, in our study with a mean follow up time of approximately 10 months, only one patient required revision ESS and only after stopping dupilumab therapy due to an adverse reaction. The ability to reduce or delay the need for ESS and subsequent revision surgeries is an important consideration in this patient population who have a high propensity for multiple surgical interventions, particularly among patients with AERD [25]. This study had a large proportion of patients with AERD (41%) and further demonstrates effectiveness in this difficult-to-treat patient population in the real world setting. Other studies evaluating the effectiveness of perioperative dupilumab have demonstrated that after revision ESS and dupilumab therapy, only one out of eight patients in their cohort developed regrowth of nasal polyps within 9 months and none required a revision surgery [26]. Reducing the need for surgery has important implications for not only individual patients but also for healthcare system spending and resource allocation. Interestingly, preliminary cost utility analysis concluded that FESS is the more cost effective strategy for the treatment of CRSwNP upfront when compared to dupilumab [27, 28]. However, longer term studies are needed, particularly in Canadian context, to determine if the higher annual cost of dupilumab therapy is offset by the reduction in revision surgeries, hospital visits, and improvements in disease-related quality of life.

Reported adverse effects associated with dupilumab therapy include worsening of nasal polyposis or asthma, eosinophilia, epistaxis, pain at the injection site, arthralgia, conjunctivitis, and nasopharyngitis [11]. In a large randomized control trial by Bachert et al., only 3% of patients receiving dupilumab (compared to 5% in placebo) experienced adverse events leading to treatment discontinuation. In the current study, only two patients discontinued therapy; one because of severe lower limb edema, which resolved with the discontinuation of dupilumab, and another because of pain at the injection site. Thus, our study further reinforces that dupilumab has a relatively low incidence of serious adverse effects, but it is certainly something that needs to be continually monitored and studied in the real-world clinical setting.

## Limitations

Our study demonstrates robust improvements in sinonasal-specific outcome measures following dupilumab therapy, however, there are several key limitations. First, given that dupilumab is a relatively novel therapy that was introduced to our clinic within the last two years, our study design does not capture long-term outcomes beyond 12 months, thereby highlighting the need for similar retrospective and prospective studies with longer follow-up durations. Given that this is a retrospective study, it is difficult to quantify patients’ use of oral corticosteroids and intranasal corticosteroids including duration and frequency of use as well as their compliance with the medications. Furthermore, this study did not include outcomes for patients’ asthma, which is an important metric as dupilumab was initially approved for severe asthma. Moreover, there was a significant time interval (31.1 months) from patients last surgical intervention to starting dupilumab which may have impacted their outcomes. The delay in initiating therapy with dupilumab is likely secondary to a multitude of factors including the fact that patients typically have improvement from surgery for approximately 1 year prior to seeking additional therapy in addition to insurance approval delays for obtaining the medication.^25^ Additionally, the validity of this study is limited by its relatively small sample size of patients. Despite the limitations described here, our study suggests that in this cohort of patients, dupilumab treatment improves sinonasal symptoms and quality of life in patients with difficult-to-treat and medically refractory CRSwNP.

## Conclusion

Among 27 patients with CRSwNP receiving dupilumab, SNOT-22 scores decreased 6.2%, 33.3%, 56.9% and 56.9% from baseline at 1, 3, 6, and 12 months, respectively. Only 2 patients discontinued dupilumab due to adverse events. The only patient who required revision FESS was one who had previously stopped dupilumab. Taken together, these findings suggest that dupilumab therapy is safe and efficacious in reducing sinonasal symptom burden, improving patient quality of life, and preventing revision FESS in patients with CRSwNP. This is one of few studies that have measured the therapeutic efficacy and assessed the accessibility of dupilumab in Canadian healthcare settings. This study further strengthens the existing body of literature supporting the addition of dupilumab as an adjunctive treatment for refractory CRSwNP, a disease entity with high symptom burden and quality of life impact that has remained historically challenging to treat.

## Data Availability

The datasets generated and analyzed during the current study are available from the corresponding author on reasonable request.
